# Nonlinear Correlation of POD and DMD Modal Coefficients in Reduced-Order Modeling of Flow Around a Cylinder in a Microchannel

**DOI:** 10.3390/mi17070778

**Published:** 2026-06-26

**Authors:** Bin Zuo, Xiaopei Yang, Haichun Wang, Qianhao Xiao

**Affiliations:** 1 School of Mechanical and Electrical Engineering, Suzhou Polytechnic University, Suzhou 215104, China; 91726@jssvc.edu.cn; 2 Jiangsu Province Robot and Intelligent Equipment Engineering Technology Research and Development Center, Suzhou 215104, China; 3 Kingclean Electric Co., Ltd., Suzhou 215011, China; 4 School of Energy and Power Engineering, Changsha University of Science and Technology, Changsha 410114, China; xiaoqh@csust.edu.cn

**Keywords:** microchannel, proper orthogonal decomposition, dynamic mode decomposition, reduced-order model, the sparse identification of nonlinear dynamics

## Abstract

Nonlinear correlations among modal coefficients enable interpretable reduced-order models (ROMs) for microfluidic flows. In this study, flow around a cylinder in a microchannel at Re = 100 is investigated using proper orthogonal decomposition (POD), dynamic mode decomposition (DMD), and POD + DMD. The sparse identification of nonlinear dynamics (SINDy) is employed to identify nonlinear correlations among modal coefficients. The results show that the first POD mode contains 33% of the total kinetic energy, and the first 14 modes capture 99.2% of the energy. A minimal ROM with only two degrees of freedom is constructed, in which the real and imaginary parts of active modal coefficients differ in phase by π/2 and their magnitude equals the vortex-shedding fundamental frequency (1.067 Hz). Among sparse regression algorithms, the FROLS method yields the sparsest representation (sparsity rate 0.05), whereas other methods give sparsity rate > 0.3. Reducing the temporal resolution from 0.01 to 0.1 increases the manifold dynamics coefficient error from 0% to 0.56%. Only the ROMs built from POD + DMD and DMD preserve essential kinematic resolution. The POD-based ROM fails to maintain correct energy levels over long-time integration. Therefore, the nonlinear correlation between POD + DMD modal coefficients is recommended for developing ROMs in microchannel flows when accuracy, interpretability, and stability are considered together.

## 1. Introduction

Many unsteady and multi-scale flow phenomena in microfluidic devices, such as cylinder wakes in microchannels, exhibit large-scale coherence that can be captured by low-dimensional attractors or nonlinear dynamic systems described by manifold equations [1,2,3]. For example, Lorenz [4] used three-dimensional ordinary differential equations (ODEs) to construct the minimal reduced-order model (ROM) of the two-dimensional Benard convection problem. The nonlinearity in the dynamic system dominates the potential spatiotemporal dynamics and modes. It does not destroy the potential low-dimensional properties [5]. Therefore, constructing the ROM is very important for mining and utilizing this low-dimensional flow, profoundly understanding the flow structure, and implementing engineering applications.

The techniques for ROM are too numerous to cite individually, emerging endlessly from physical simplification to data-driven approaches. Among them, the pioneering work of Aubrey et al. [6] has extensively promoted the development of ROM. The most energetic modes are extracted by proper orthogonal decomposition (POD) [7,8,9], which facilitates obtaining low-dimensional ordinary differential equations from Navier–Stokes equations through Galerkin projection, deepening the understanding of the coherent structure of the wall region of the turbulent boundary layer. Equally influential is the work of Noack et al. [10] in constructing a low-dimensional model hierarchy for flow around a cylinder. The introduction of shift mode to represent low-energy modes significantly improves the resolution of transient dynamics. Tadmor et al. [11] proposed the transient modal energy flow analysis method and deeply discussed shift mode’s contribution to improving ROM stability and simplicity. For more information on the methods and applications of ROMs in related fields such as fluids, please refer to the review articles of Brunton et al. [12,13], Taira et al. [14], and Ahmed et al. [15].

Nonlinearity is widely studied in energy applications, such as discovering correlations [16,17,18], solving optimization problems [19,20], and forecasting [21]. When evaluating the complexity of low-rank approximation, complex systems such as flow face the challenge of high-order nonlinearity. The empirical interpolation method (EIM) [22] and simplified discrete empirical interpolation method (DEIM) [23] were used in the POD to overcome the complexity caused by nonlinearity. The former is a computationally effective sparse sampling method to solve nonlinear terms. The latter is constructing a learned POD modal library and using compressed sensing to identify the active POD subspace required by the low-dimensional POD–Galerkin model [24,25,26]. Sargsyan et al. [5] and Mohebujjaman et al. [27] combined the two strategies to characterize the fluid flow. They realized the pressure field reconstruction around the cylinder at different Reynolds numbers. The success of this method lies in that the POD library can classify the dynamic characteristics robustly.

Rowley and Dawson [28] pointed out that the POD ignores the time correlation and does not consider the low-energy mode, significantly affecting the flow dynamic characteristics. Although all the energy is captured, it often produces poor low-dimensional models. At the same time, the truncation error will also lead to the instability of the ROM [29,30,31]. Carlberg et al. [32] and Grinberg et al. [33] discussed this in depth from a numerical point of view. Balajewicz [34] modified the eddy viscosity on the classical turbulence closure model, and Mohebujjaman [35] proposed an alternative Galerkin scheme to overcome the truncated problem.

Different from the above nonlinear solution strategy, Callaham et al. [36] combined the approach of POD, dynamic mode decomposition (DMD) [37,38], and the sparse identification of nonlinear dynamics (SINDy) [39], and constructed a ROM by using the nonlinear correlation in modal coefficients in cavity flow. The key to success is that the DMD of POD modal coefficients will make the coefficients closer to the form of pure oscillation, which is convenient to learn a compact and interpretable dynamic system model from the active modal coefficients by using SINDy. This method captures the correct energy cascade, and the ROM does not need a closure hypothesis to be more stable and robust than the POD–Galerkin model.

**Figure 1:**
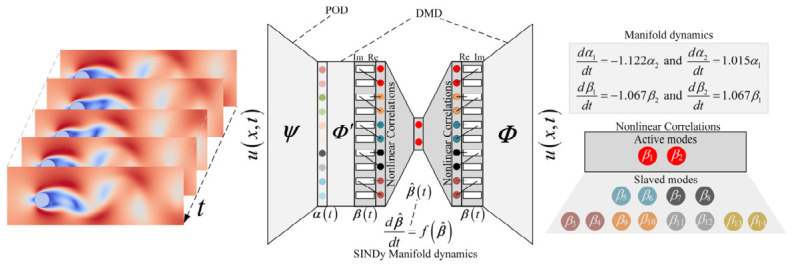
Schematic diagram of building a ROM. Re and Im represent the real and imaginary parts of the coefficients, respectively.

A ROM of laminar flow around a cylinder was constructed in this work to explore more details of the Callaham strategy [36]. Figure 1 shows that the POD and DMD determine the three modal coefficients. The nonlinear correlation of different modal coefficients was used to construct manifold dynamics. The ROM based on POD modal coefficients has two degrees of freedom. The modal coefficients always appear in pairs, and a simple polynomial function can accurately express the nonlinear correlation. The manifold dynamics system constructed by SINDy consists of a couple of active modes whose coefficients are the fundamental frequency *f*_1_ of vortex shedding. An important innovation of this study is the discovery that constructing accurate ROMs requires high temporal resolution and is highly dependent on the nonlinear correlation of high-energy modes.

## 2. Simulation of Flow Field

**Figure 2:**
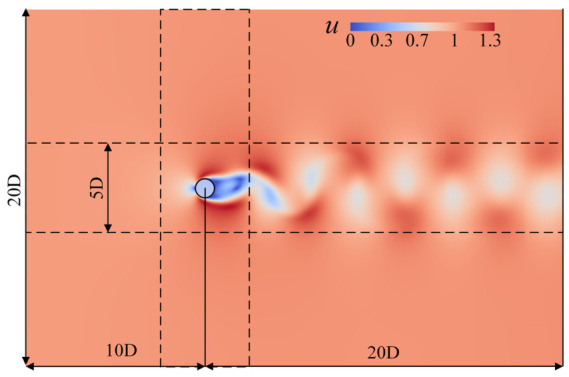
Fluid domain and wake of flow around a cylinder.

The flow considered in this work is the laminar flow around a cylinder confined in a microchannel (Figure 2). Despite more than a century of in-depth studies on cylinder flow, during which transient characteristics such as two-dimensional periodic vortex shedding for 50 < Re < 175 and the transition to turbulence have been well understood, the nonlinear correlations of modal coefficients are rarely reported in microchannels. The computational domain is non-dimensionalized by the cylinder diameter D = 1 for generality, corresponding to a physical microchannel with D = 100 μm (channel width 20D = 2 mm, length 30D = 3 mm. This scale is typical for lab-on-a-chip and microfluidic heat sink applications [40]. At Re = 100, the flow remains laminar and exhibits periodic vortex shedding, similar to macroscopic flows. The fluid domain is divided by a structured grid, and the mesh in the 5D region around the cylinder is refined.

It is well known that the incompressible Navier–Stokes equation describes the flow state of flow around a cylinder in a laminar state:

(1) $$\begin{array}{c} \frac{\partial u}{\partial t}+\nabla \cdot \left(u⊗u\right)=-\nabla p+\frac{1}{Re}\nabla^{2}u\\ \nabla \cdot u=0 \end{array}$$

where ***u*** and *p* are the velocity field and pressure field, respectively.

The computational fluid dynamics (CFD) software Ansys Fluent 2024 was used to obtain the velocity field, using the finite volume method (FVM) to solve the Navier–Stokes equation. The Inlet and Outlet boundaries are Velocity Inlet and Outflow, respectively. The incoming flow velocity U is 1. Symmetrical boundary conditions [41,42,43] are arranged on both sides of the fluid domain, and no-slip wall boundary conditions are used for the cylinder. The flow field was solved using a laminar flow model. Spatial discretization employed the second-order upwind scheme, while temporal discretization was handled with the second-order implicit formulation. The pressure–velocity coupling was resolved via the SIMPLE algorithm. Convergence was assumed when all residuals for continuity and the x- and y-momentum equations fell below 1 × 10^−6^. The time resolution is Δ*t* = 0.01, and the sampling frequency is about 600 times the vortex-shedding frequency. The reason for the higher sampling frequency compared to other studies will be discussed in Section 5. A total of 20,000 time steps were calculated, and the last 10,000 snapshots were retained for analysis.

According to the definition of the Strouhal number (St = *f_v_D*/*U*), *f_v_* denotes the vortex-shedding frequency. To verify grid independence, a detailed comparison of the Strouhal number and the cumulative energy fractions of the first five POD modes (see Section 3.1) was conducted across five different grid resolutions (Table 1). As the grid count increased from 12,000 to 160,000, the maximum deviations in St and POD energy fraction relative to the finest grid were 4% and 5.3%, respectively. For the grid with 100,000 cells, the deviations reduced to only 0.6% and 0.35%. These results indicate that further grid refinement has a negligible impact on the global vortex-shedding frequency and the dominant modal energy distribution of interest. Accordingly, considering both computational cost and adequate POD energy resolution, the case of flow past a circular cylinder in a microchannel with 100,000 cells was selected for detailed analysis. In this study, the vortex-shedding frequency (*f_v_* = 0.17) is consistent with the results of Kim [41], Shen [42], and Park [43], verifying the validity of the numerical method used.

Sipp and Lebedev [44] studied the stability of the mean flow around the cylinder and the cavity flow through a global weakly nonlinear analysis. The unstable mean flow has limited relevance and usefulness in the statistically stationary state. Therefore, decomposing the velocity field into the time-averaged flow $\bar{u}$(*x*) and the pulsating velocity *u*′(*x*, *t*) is more relevant. Then the pulsating velocity and its pulsating kinetic energy are defined as

(2) $$\begin{array}{c} u\left(x,t\right)=\bar{u}\left(x\right)+u^{\prime }(x,t)\\ E\left(t\right)=\frac{1}{2}\int_{\Omega }u^{\prime }\left(x,t\right)\cdot u^{\prime }\left(x,t\right)d\Omega \end{array}$$

where *Ω* is the fluid domain.

## 3. Modal Analysis

Modal analysis solves the complexity of flow characteristics in time and space by finding the critical features (modes) of complex flow to promote understanding of complicated flow behavior. A ROM can be constructed based on modal analysis to obtain the flow structure with significantly reduced computational cost. Still, it can also be used in studies such as aerodynamic optimization design [45,46,47] and flow control [48,49,50]. Taira et al. [14] have comprehensively described modal analysis techniques in recent decades, and here we only give a brief introduction to POD and DMD.

### 3.1. Proper Orthogonal Decomposition

As the initial modal analysis method, proper orthogonal decomposition was introduced by Lumley [7] in the context of solving turbulence and complex space–time field problems. It decomposes the high-order nonlinear flow field into several spatial orthogonal modes and ranks them according to the modal energy from large to small. POD is vital in analyzing flow mechanisms such as an impinging jet on a slotted plate [51].

Since POD can be regarded as a continuous form of singular value decomposition [52,53], this study directly utilizes singular value decomposition to obtain a POD basis.

(1) The velocity field in the flow around the cylinder is sampled with constant time resolution, and then a large dataset X = [*u*_1_, *u*_2_, …, *u_m_*] consisting of the snapshot matrix *u_k_* is obtained.
(2) Dataset X is used for singular value decomposition (X = UƩV*) to obtain the left singular value vector U, singular value Ʃ, and right singular value vector V. Low-rank truncation is performed according to the distribution of singular value energy. The first r order of the left singular value vector truncation is used as the POD basis $\tilde{U}$ = [*ψ*_1_, *ψ*_2_,…, *ψ*_r_]. The modal coefficient is *α* = [*α*_1_, *α*_2_,…, *α_r_*].

**Figure 3:**
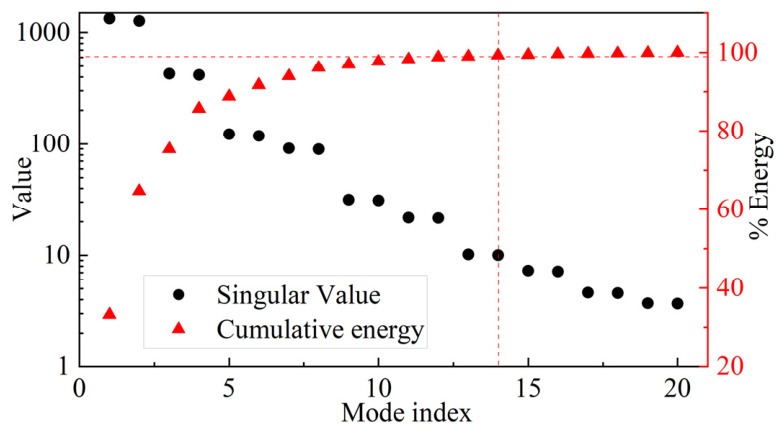
Singular value spectrum and cumulative energy distribution.

POD was applied to the pulsating velocity field, and its singular value spectrum and cumulative energy distribution are shown in Figure 3. The singular value converges relatively fast. The first mode contains 33% of the total energy, and the first 20 modes account for 99.99%. At order *r* = 14, 99.2% of the energy is recovered, so the first 14 modes and modal coefficients are used for further analysis.

**Figure 4:**
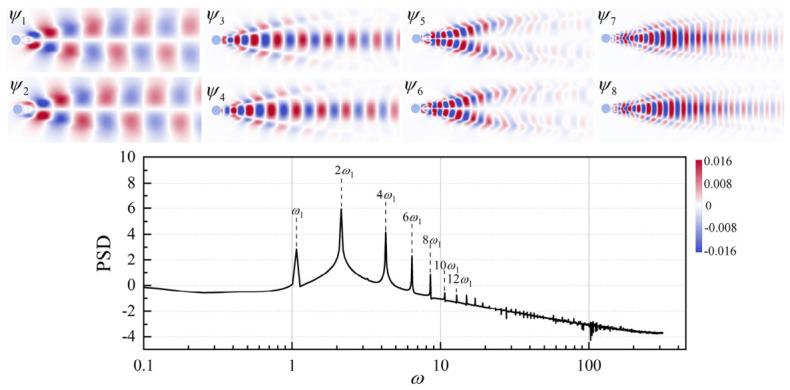
The first eight modes of POD and the power spectral density of modal coefficients.

As shown in Figure 4, the POD modes of pulsating velocity appear approximately in pairs. The modes are antisymmetric up and down along the *x*-axis. The alternating symmetry properties of the POD modes in this study are consistent with Deane et al. [54], Ma and Karniadakis [55], and Noack et al. [10]. The power spectral density (PSD) of modal coefficient shows that the modal coefficient captures the fundamental frequency and frequency doubling of vortex shedding but ignores the odd times. The fundamental frequency of vortex shedding is ω_1_ = 2π*f_v_* = 1.067.

### 3.2. Dynamic Mode Decomposition

DMD is a modal analysis method introduced by Schmid [38], which can capture the coherent flow structure and contain dynamic information. It provides a spatial correlation structure in which each mode has the same linear behavior in time, which means that DMD offers a set of modes to simplify the flow and provides how the modes evolve with time. Ali and Guo [56] used DMD to evaluate underground natural dynamic pressure of gas reservoirs quickly.

**Figure 5:**
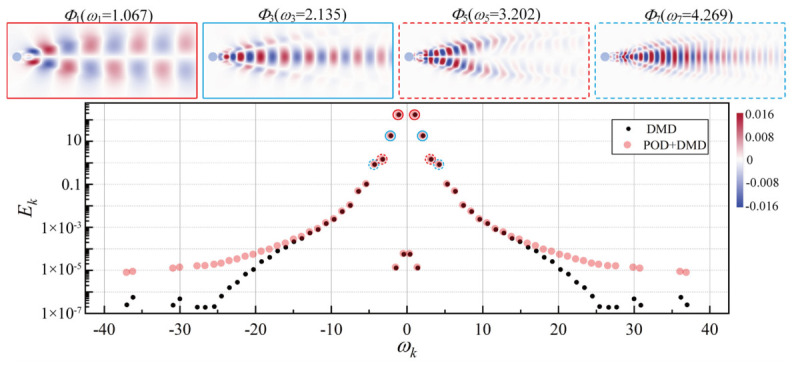
The velocity distributions corresponding to the real part of the first four pairs of high-energy modes of the DMD.

This study follows the exact DMD algorithm given by Tu et al. [57]. As shown in Equation (5), through the singular value decomposition of matrix X = [*u*_1_, *u*_2_, …, *u_n_*_−1_], the spectral decomposition of approximate matrix $\tilde{A}$ is obtained to approximate the DMD mode (Figure 5).

(3) $$\begin{array}{l} X\approx \tilde{U}\tilde{\Sigma }{\tilde{V}}^{*}\\ \tilde{A}={\tilde{U}}^{*}X^{\prime }\tilde{V}{\tilde{\Sigma }}^{-1}\\ \tilde{A}\;W=W\;\Lambda \end{array}$$

where *W* is the eigenvector matrix of the approximate matrix $\tilde{A}$, and X′ is the time-shift matrix (X′ = [*u*_2_, *u*_3_, …, *u_n_*]). The diagonal entries *λ* of the diagonal matrix Λ are the eigenvalues of the DMD, and each column ω of W is the eigenvectors of A ~. The DMD mode φ corresponding to the eigenvalue λ is

(4) $$\varphi =\frac{1}{\lambda }X^{\prime }\tilde{V}{\tilde{\Sigma }}^{-1}\omega$$

Similar to POD, the pulsation velocity can be written as a linear combination between the DMD mode Φ(x) and the coefficient *β*(*t*).

Low-rank truncation of the POD retained the first 20 modes, preserving 99% of the total energy. For the DMD, truncation was applied at the 64th mode. To compare the resulting mode characteristics, Figure 5 presents the energy distribution of the DMD modes as a function of frequency. Notably, unlike the Fourier spectrum of the POD temporal coefficients, which captured only even-order harmonics, the first 14 DMD modes exhibited frequencies that corresponded precisely to the fundamental vortex-shedding frequency and its higher harmonics. Furthermore, comparing the modal structures obtained from POD and DMD indicates that while POD primarily identifies large-scale coherent structures in the flow field, DMD resolves more detailed dynamical features.

### 3.3. POD + DMD

Callaham et al. [36] verified the ability to identify potential attractors from POD modal coefficients from a square cavity flow. The key is to perform DMD on the POD modal coefficients so that the projected modal coefficients are closer to pure oscillation. In this process, the role of DMD is not as a tool for dimensionality reduction but to launch the POD modal coefficients to pure oscillation whose coefficients approximate a single frequency. The dimension of POD modal coefficients is so tiny that the discrete-time linear evolution operator *A* and its spectral decomposition can be calculated directly. Then the POD modal coefficient is expressed as

(5) $$\alpha \left(t\right)=\sum_{k=1}^{r}\Phi_{k}\left(x\right)\beta_{k}\left(t\right)$$

**Figure 6:**
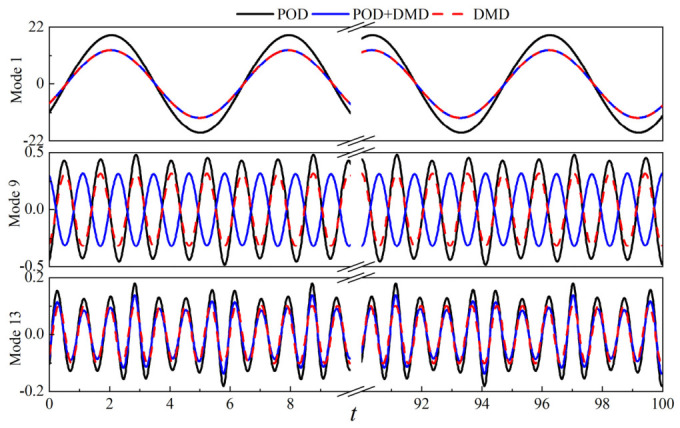
Comparison of three modal coefficients. The POD + DMD and DMD show the real part of the modal coefficient.

The POD modal coefficients are shown in Figure 6 after dynamic modal decomposition. In the first fourteen pairs of modes, the modal coefficients’ average kinetic energy and frequency distribution are consistent with the DMD. The average pulsating kinetic energy of POD + DMD is generally higher than that of DMD in the high-frequency part.

The first nine orders of POD modal coefficients are all in the form of pure oscillation (Figure 6). After dynamic modal decomposition, the modal coefficients of the 13th order are in the form of impure oscillation. The superiority of DMD makes these 14 modal coefficients appear to be pure oscillation form. In addition, POD + DMD coincides with some modal coefficients of DMD, such as the first modal coefficient.

## 4. Reduced-Order Model

The construction of the ROM has achieved significant results in revealing the low-dimensional flow structure and significantly reducing the computational cost of reconstructing the flow field. It is well known that the POD–Galerkin method [6,9] and sparse sampling of the space [22,23], which project a high-dimensional flow field into a low-dimensional subspace, significantly contribute to the success of building reduced-order models. Callaham et al. [36] proposed that the method of constructing a ROM without closure and equation is equally attractive. This strategy is briefly introduced in this section.

### 4.1. Nonlinear Correlation

**Figure 7:**
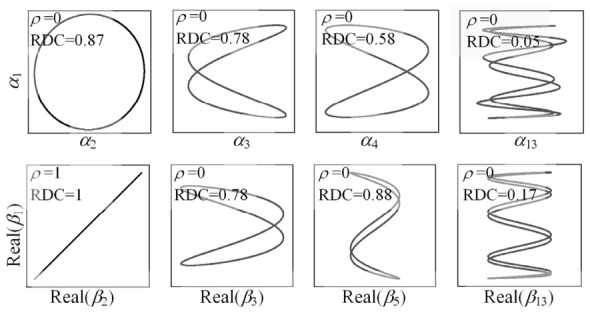
Measurement of linear and nonlinear correlation of POD and DMD coefficients.

Lopez-Paz et al. [58] introduced the randomized dependence coefficient (RDC) to measure nonlinearity between random variables of arbitrary dimensions. RDC is empirically valid and scalable to large datasets like modal coefficients. Figure 7 shows that the POD coefficients are orthogonal, and the coefficients are uncorrelated in a linear sense (ρ = 0). It does not mean that the coefficients are uncorrelated in a nonlinear sense (RDC ≠ 0).

There is an apparent functional relationship between modal coefficients, such as POD modal coefficients *α*_1_, *α*_2_, and *α*_3_. It is also implied by the pulsating velocity distribution of the mode. Callaham et al. [36] pointed out that the coefficients can be accurately approximated by a simple algebraic function, whether linear or nonlinear. According to the functional relationship between the coefficients, the modal coefficients can be divided into active and slaved modal coefficients. Once the functional relationship is determined, the active mode can represent the whole modal coefficient.

**Figure 8:**
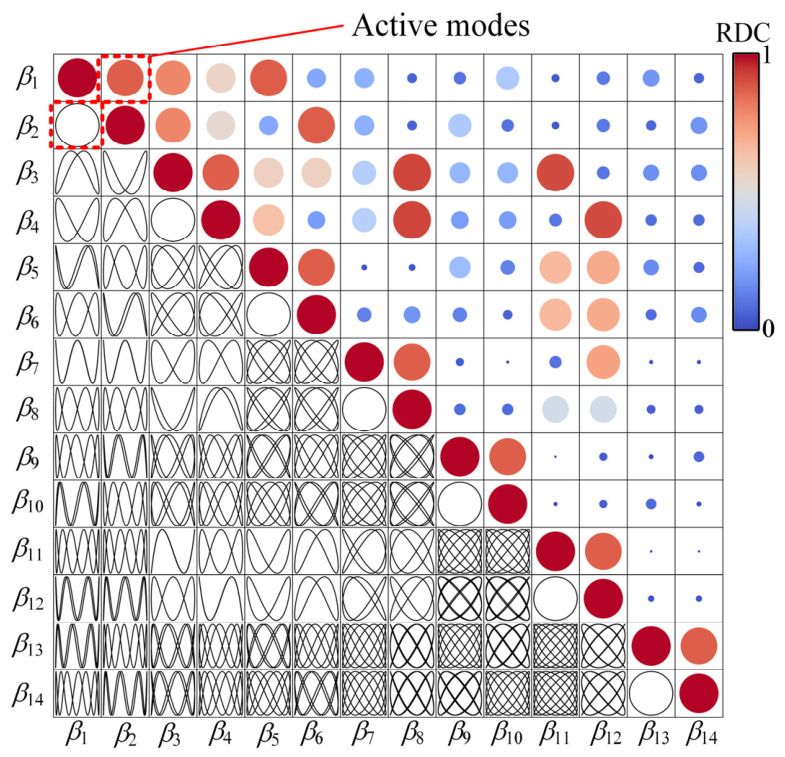
Nonlinear correlation measurements of DMD modal coefficients.

Since the modal coefficients of POD + DMD and DMD exist in complex conjugate pairs, a pair of conjugate modal coefficients can be represented by the real and imaginary parts of the first modal coefficient. As shown in Figure 1, the nonlinear correlation between modal coefficients is constructed by extracting the real and imaginary parts of each pair of modal coefficients to form a matrix of the same dimension as the original modal coefficients. Figure 8 presents the Lissajous figure and RDC values after transforming the DMD modal coefficients. The RDC value between a pair of DMD modal coefficients (*β*_1_ and *β*_2_, *β*_3_ and *β*_4_, etc.) is enormous. The Lissajous figure is approximately circular, so the active modal coefficients appear in pairs. There is also a robust nonlinear correlation between the high-energy modal coefficients and the low-energy modal coefficients, such as (*β*_3_, *β*_11_) and (*β*_4_, *β*_12_).

### 4.2. The Sparse Identification of Nonlinear Dynamics

Although the RDC value measures the nonlinear correlation between the modal coefficients, it does not directly reflect the causal relationship between the modal coefficients. It also seems complicated to determine whether the corresponding modal coefficients are active modes from the POD mode and the DMD mode. From the active modes constructed by Callaham et al. [36], high-energy modal coefficients and coefficients with large RDC are more likely to be active modal coefficients. A simple and effective method is to assume a group of active modes, construct the functional relationship, and then reconstruct the whole modal coefficient by constantly observing the reconstruction error of the slaved mode until the most suitable active mode is found. So, constructing a set of algebraic functions to represent the relationship between modal coefficients has become the key.

Brunton et al. [39] proposed the sparse identification of nonlinear dynamics model to identify the governing equations. The SINDy model is an equation-free data-driven approach. The key is building the library and solving sparse matrices. Therefore, it is possible to construct either functional relationships between multiple variables or ordinary differential equations depending on the library. The method can also utilize the partial differential library to discover the Navier–Stokes equations [59]. The SINDy model is given a brief introduction in this section.

For a dynamic system generally expressed by ordinary differential equations, it can be written as

(6) $$\frac{d}{dt}x\left(t\right)=f\left(x\left(t\right)\right)$$

where *x*(*t*) represents the state vector varying with time *t*, and function *f* represents the motion equation of the dynamic system. As we all know, function *f* consists of only a few terms for many complex systems such as fluids. The X matrix is very sparse. SINDy gives an equation-free method to solve the equation of motion *f*:

(7) $$\dot{Χ}=\Theta \left(Χ\right)\Xi$$

The matrix $\dot{X}$ column represents the derivative of state x (*t*) to time *t*. Θ(X) is a nonlinear relation library composed of each state. Each column of Ξ represents a sparse vector. When Ξ is determined, the governing equation of each row can be constructed as follows:

(8) $$\frac{d}{dt}x_{k}\left(t\right)=f_{k}\left(x\left(t\right)\right)=\Theta \left(x{\left(t\right)}^{T}\right)\xi_{k}$$

The core of the SINDy model is to build an appropriate library and solve sparse matrices. After determining the library, solving the sparse matrix is equivalent to solving the following nonconvex optimization problem:

(9) $$\underset{\Xi }{\mathrm{minimize}}{{\left\|\left.\dot{Χ}-\Xi \Theta \left(Χ\right)\right\|\right.}_{2}}^{2}+\gamma {\left\|\Xi \right\|}_{0}$$

where *γ* is weight regularization. Brunton uses the least absolute shrinkage and selection operator (LASSO) to solve the sparse matrix in the original text and gives an alternative scheme based on sequential thresholded least squares (STLSQ) when facing the problem of a massive amount of data.

The model method of finding governing equations from a large number of data in the SINDy model has attracted much attention, especially the problem of solving sparse matrices. Boninsegna et al. [60] introduced the cross-validation method and proposed a stepwise sparse regressor (SSR) strategy. Champion et al. [61] proposed a sparse relaxation regularized regression (SR3) method, and Loiseau [62] proposed a greedy forward regression orthogonal least squares (FROLS) method, etc.

### 4.3. Construct Nonlinear Correlation

The SINDy model with a second-order polynomial library was used to establish the algebraic function relationship between active and slaved mode coefficients. Through a simple attempt, the order corresponding to the active modal coefficient in the three modal coefficients is (1,2,5,6,7,8). The angular frequencies corresponding to POD + DMD and DMD active modal coefficients are (*ω*_1_, 3*ω*_1_, and 4*ω*_1_). Although the frequencies between active modes are multiple, the nonlinear correlation is minimal.

When using STLSQ, SSR, SR3, and FROLS to solve the sparse matrix Ξ of the second-order polynomial library Θ(X), not all the sparse matrices Ξ solved are sparse. For this reason, it is vital to reflect the sparsity by the proportion of non-zero entries in the solved sparse matrix Ξ to the dimension of the sparse matrix. The sparsity rate *η* is defined as follows:

(10) $$\eta =\frac{\sum_{i=1}^{n}N(\xi_{i}\neq 0)}{nN_{Lib}}$$

where *n* is the number of slaved modes, and *N_Lib_* is the dimension of the polynomial library. When the library order is 2, 3, and 4, *N_Lib_* is 27, 83, and 209, respectively. *N*(*ξ_i_* ≠ 0) represents the number of coefficients that are not zero in the ith column of the sparse matrix Ξ. By definition, the smaller the sparsity rate, the sparser the sparse matrix to be solved.

**Figure 9:**
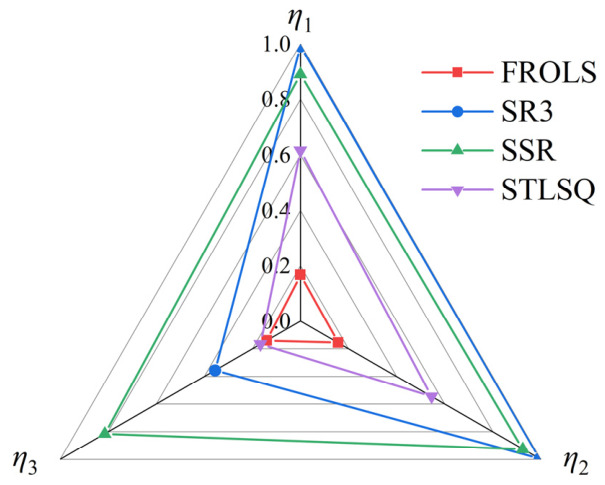
Sparse rates for different solving algorithms. The subscripts 1, 2, and 3 of *η* represent the sparse rate of POD, POD + DMD, and DMD corresponding to the sparse matrix Ξ, respectively.

Figure 9 shows that the FROLS algorithm is more suitable for solving sparse matrices and constructing nonlinear correlations between modal coefficients. When establishing the functional relationship between POD coefficients, SR3, SSR, and STLSQ almost lose sparsity. The greedy forward selection mechanism of FROLS is better suited to regression tasks on algebraic manifolds. In contrast, sparsity-penalized methods such as STLSQ, SSR, and SR3 tend to suffer from coefficient interference when handling nonlinear mappings between modal coefficients. Specifically, an excessively large threshold discards weak but meaningful contributions, whereas an overly small threshold introduces excessive noise terms. As a result, these methods struggle to strike a balance between sparsity and accuracy, often resulting in a high sparsity ratio. From the perspective of sparsity, compared with POD and POD + DMD, the functional relationship between modal coefficients of DMD is simpler, mainly because the modal coefficients of DMD are closer to pure oscillation.

Although periodic dynamics with energy conservation are described, small perturbations cause the system to adapt to new periodic dynamics at higher or lower energy levels. The ROM consisting of six degrees of freedom is specific [63,64,65]. A polynomial function relationship is established separately for the six active modal coefficients. When the FROLS algorithm is selected, and the order of the library is four, the modal coefficients (*β*_5_, *β*_6_, *β*_7_, *β*_8_) of POD + DMD and DMD can be represented by the modal coefficients (*β*_1_, *β*_2_). Equation (11) shows the polynomial function relationship between the DMD modal coefficients. Only one pair of actual active modes exists, and a ROM with two degrees of freedom is constructed. Such results are consistent with the minimal ROM dimensionality of Noack et al. [10] and Loiseau et al. [63,64]. Due to the complex polynomial function relationship, three pairs of active modes were mistakenly identified using SINDy.

(11) $$\begin{array}{l} \beta_{5}=-5.2e^{-4}{\beta_{1}}^{3}-3.9e^{-4}{\beta_{1}}^{2}\beta_{2}+1.6e^{-3}\beta_{1}{\beta_{2}}^{2}+1.3e^{-4}{\beta_{2}}^{3}\\ \beta_{7}=3.1e^{-5}{\beta_{1}}^{4}+1e^{-6}{\beta_{1}}^{3}\beta_{2}-1.9e^{-4}{\beta_{1}}^{2}{\beta_{2}}^{2}+3.1e^{-5}{\beta_{2}}^{4} \end{array}$$

**Figure 10:**
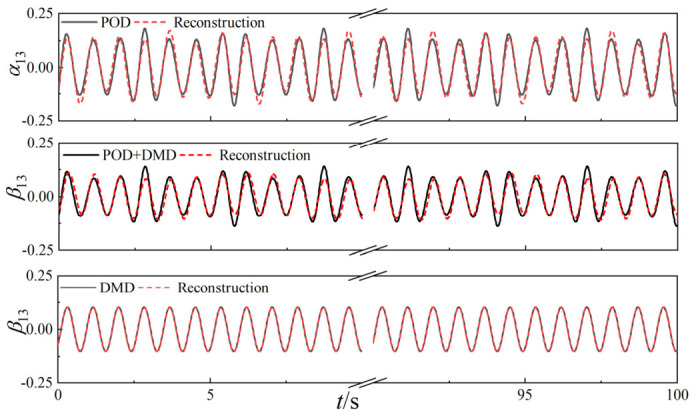
Comparison of reconstruction effects of three modal coefficients.

Only one pair of active modal coefficients shows that the nonlinear correlation between modal coefficients can easily be classified as pure harmonics [36]. The nonlinear correlation between the modal coefficients is well defined, and the slaved modal coefficients are easily reconstructed using the active modal coefficients. Figure 10 shows that the non-pure oscillation form of the last two pairs of modal coefficients of POD and POD + DMD makes the reconstruction effect unsatisfactory. In contrast, the DMD slaved modal coefficients achieve an accurate reconstruction effect.

### 4.4. Manifold Dynamics

The SINDy model was used to build an ordinary differential equation system composed of active modal coefficients to obtain an interpretable ROM. When the order of the polynomial library is one, the two-dimensional manifold can be constructed for active modal coefficient:

(12) $$\begin{array}{l} \frac{d\alpha_{1}}{dt}=-1.122\alpha_{2}\;\mathrm{and}\;\frac{d\alpha_{2}}{dt}=1.015\alpha_{1}\\ \frac{d\beta_{1}}{dt}=-1.067\beta_{2}\;\mathrm{and}\;\frac{d\beta_{2}}{dt}=1.067\beta_{1} \end{array}$$

Manifold dynamics constructed based on POD + DMD and DMD modal coefficients show that the time derivatives of the modal coefficients’ real and imaginary parts are precisely the corresponding modal coefficients’ imaginary and real parts. The absolute value of the coefficients is the vortex-shedding fundamental frequency. This characteristic is consistent with the sine and cosine functions in the trigonometric function, indicating that the difference between the real part and the imaginary part of the modal coefficient is π/2. There is no similar physical explanation for manifold dynamics constructed based on POD modal coefficients.

**Figure 11:**
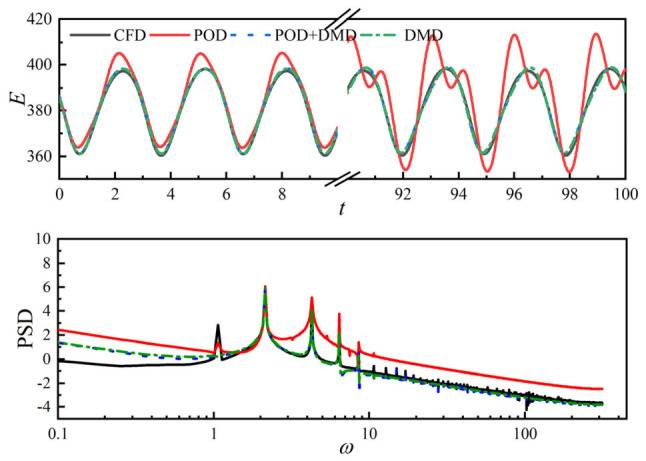
Reconstruction effect of modal coefficients. The variation in the modal coefficient pulsating kinetic energy with time (**top**). The bottom is the PSD of the modal coefficients (**bottom**).

After obtaining the ROM, it is crucial to verify the reconstruction effect of the whole velocity field. Firstly, the active modal coefficients are reconstructed by manifold dynamics. The slaved modal coefficients are reconstructed by nonlinear correlation, and finally, the whole flow field is reconstructed by mode and modal coefficients. Figure 11 shows that POD + DMD and DMD can keep the pulsating kinetic energy of the coefficient at the correct energy level. Still, POD gradually deviates from the right energy level with time. The reconstruction of the PSD shows that the three modal coefficients can capture the discrete peak in the power spectrum. Still, the POD modal coefficient is too significant to reconstruct the pulsating kinetic energy. In comparison, the reconstruction effect of POD + DMD and DMD is better.

Although POD modes are optimal with respect to energy, their temporal coefficients do not exhibit single-frequency behavior (Figure 10). Consequently, SINDy manifold regression must introduce additional higher-order nonlinear terms to fit these non-purely oscillatory waveforms, which subsequently induce a zero-frequency drift during time integration. In contrast, POD + DMD and DMD modes are dynamic; their temporal coefficients are nearly purely oscillatory (single-frequency) and evolve according to simple harmonic motion. The manifold dynamics under the SINDy framework (Equation (12)) essentially correspond to a simple harmonic oscillator with a pair of conjugate pure imaginary roots, representing the vortex-shedding flow in the microchannel. Because the POD mode coefficients contain multiple superimposed frequencies, their manifold regression is more susceptible to interference from higher-order nonlinear terms, resulting in energy leakage into unresolved high-frequency components during integration.

Noack et al. [10] explicitly stated that a low-dimensional reduced-order model (ROM) for flow past a circular cylinder must incorporate a shift mode to capture the mean-flow correction. POD retains only the fluctuating modes and discards the evolution information of the mean flow, which results in a slow drift of the oscillation center of the active modes in the manifold equations. The POD + DMD approach implicitly compensates for this limitation through the spectral projection process of DMD. The purely oscillatory nature of POD + DMD and DMD transforms the modal coefficients into an approximately single-frequency, purely oscillatory form. This property enables SINDy to accurately identify the standard simple harmonic manifold equation (Equation (12)) without introducing additional nonlinear correction terms, thereby preserving energy conservation during long-term integration.

**Figure 12:**
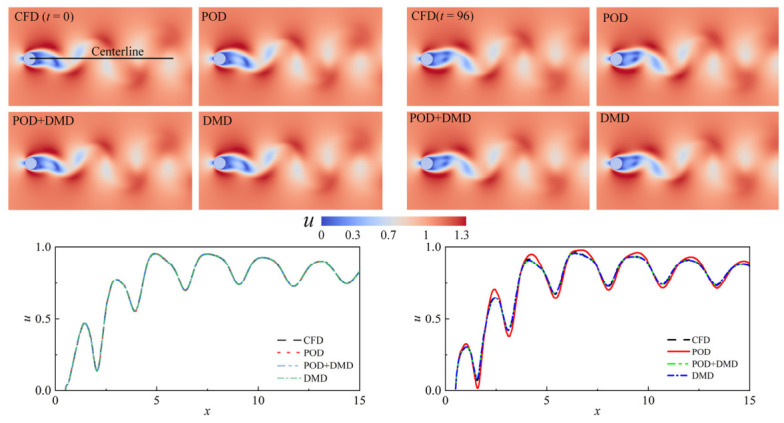
Reconstruction and comparison of the velocity field at different times.

Figure 12 shows the velocity field reconstruction effect at different times. Time *t* = 0 indicates that the reconstruction of the POD modal coefficients captures the correct energy levels, while time *t* = 96 suggests shifting the proper energy levels. Regardless of whether the reconstruction can maintain the appropriate energy level, the reconstruction of the three can reflect the correct flow field structure. The distinction between the three reconstructed velocity distributions and the vortex-shedding structure is challenging to find. Velocities on the cylinder centerline were counted to further search for differences in reconstructions. When the energy level is correctly captured, the velocity distribution on the centerline of the cylinder is the same. Still, when the POD cannot capture the correct energy level, the reconstruction results of POD are significantly different from the other two. Therefore, only the reconstruction effect of POD + DMD and DMD can maintain the correct kinematic resolution. In conclusion, POD + DMD and DMD should be recommended to investigate the nonlinear correlation between modal coefficients and construct interpretable manifold dynamics.

## 5. Time Resolution

The time resolutions were Δ*t* = 0.1 and Δ*t* = 0.05, respectively, and were used to study the effect of different time resolutions on the establishment of ROM. A total of 2000 and 4000 snapshots were saved on the same mesh configuration after the lift kept periodically fluctuating. The total time is 200, equivalent to 34 times of vortex shedding.

Table 2 presents the Strouhal number and the cumulative energy fractions of the first five POD modes at various temporal resolutions. Across the three temporal resolutions, the maximum deviation in the Strouhal number is 0.6%, and the maximum deviation in the cumulative energy fractions of the first five POD modes is 0.5%. These results indicate that the simulations of flow past a cylinder at the examined temporal resolutions exhibit strong numerical convergence, demonstrating that the CFD solution is effectively independent of the selected time step.

(1) Effects on manifold dynamics

**Figure 13:**
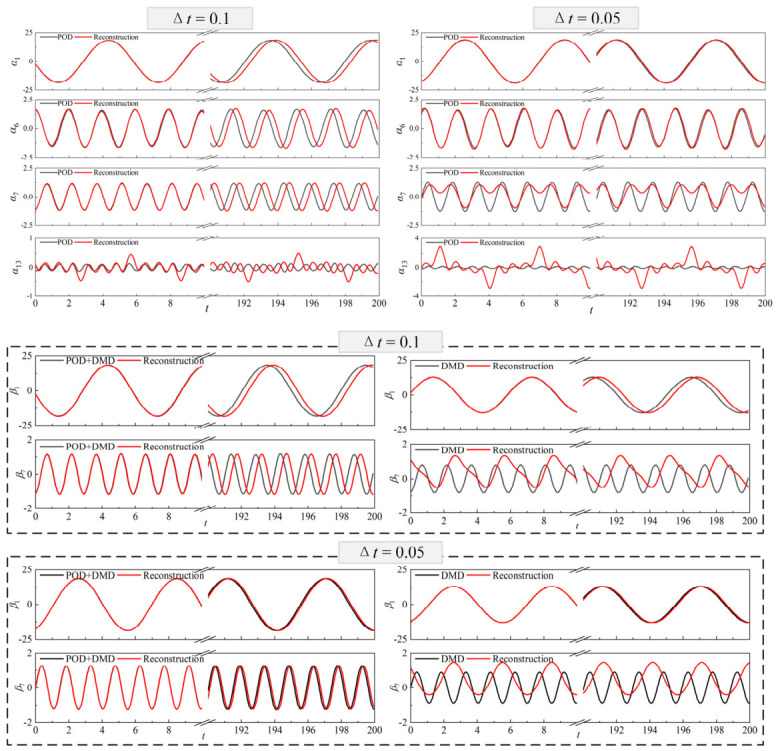
Reconstruction effect of modal coefficients at different time resolutions. The POD (**top**). The DMD and POD + DMD (**bottom**).

The time resolution significantly affects the manifold dynamics constructed based on POD modal coefficients, not only the coefficients but also the formal differences (Equation (13)). It is usual for the positive and negative coefficients to appear reversed in a two-dimensional manifold due to the different origins of the velocity fields. Figure 13 shows the reconstruction effect of the POD modal coefficients. As the time resolution decreases, the reconstruction accuracy of the modal coefficients (*α*_7_, *α*_8_) does not improve. Therefore, only when the time resolution is small enough the POD modal coefficients can be used to construct more accurate manifold dynamics.

(13) $$\begin{array}{l} \Delta t=0.1:\left\{\begin{array}{c} \frac{d\alpha_{1}}{dt}=0.004\alpha_{1}\;-1.119\alpha_{2}\\ \frac{d\alpha_{2}}{dt}=1.006\alpha_{1}-0.004\alpha_{2} \end{array}\right.\\ \Delta t=0.05:\left\{\begin{array}{c} \frac{d\alpha_{1}}{dt}=1.121\alpha_{2}\\ \frac{d\alpha_{2}}{dt}=-1.013\alpha_{1} \end{array}\right. \end{array}$$

Table 1 shows the effect of different time resolutions on the minimal ROM. The error of the ODE coefficients decreases as the time resolution decreases, and the reconstruction effect of a pair of active modal coefficients corresponding to Figure 13 is more and more accurate. Since the two forms of constructing manifold dynamics are consistent, the reconstruction effect of a pair of active modes is also highly compatible. The 0.56% error reported in Table 3 represents the maximum identification error of the frequency coefficients of the active modes in the manifold dynamics equation. At Δt = 0.1, the SINDy-identified frequencies of the active modes are ±1.061, yielding a relative deviation of only 0.56% from the true fundamental frequency ω_1_ = 1.067. This indicates that even at a relatively coarse temporal resolution, the oscillation frequency of the high-energy active modes can still be accurately captured, and the conjugate pure imaginary root structure of the manifold equation is fully preserved.

(2) Effects of nonlinear correlation

The slaved modal coefficients are easily reconstructed from the reconstructed active modal coefficients and nonlinear correlations. As the time resolution decreases, the accuracy of the modal coefficient reconstruction does not improve. The modal coefficient *α*_13_ deviates even further from the correct energy level. Therefore, the modal coefficients can be accurately reconstructed using the modal coefficients of the POD only if the temporal resolution is sufficiently small.

Although the manifold dynamics of the build remain consistent, the reconstruction effect of POD + DMD modal coefficients is significantly better than that of DMD modal coefficients. The reconstruction error of the POD + DMD modal coefficients is only reflected in the phase, and its accuracy increases continuously with the decrease in the time resolution. For DMD modal coefficient *β*_7_, neither peak nor phase is captured after reconstruction, and there is no significant improvement with decreasing time resolution. Therefore, for the DMD modal coefficients, minor differences in the constructed manifold dynamics can also seriously affect the accuracy of the ROM.

**Figure 14:**
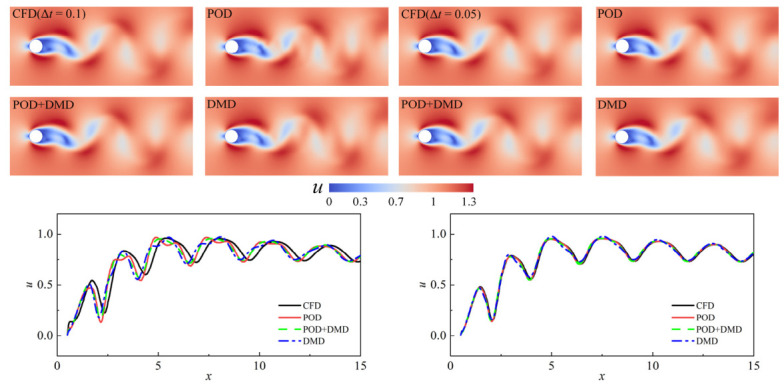
The reconstruction effect of the minimal ROM in the velocity field of *t* = 198.

The minimal reduced-order model constructed from the three sets of modal coefficients yields only minor differences in the reconstructed velocity field, as shown in Figure 14, and the velocity distribution along the cylinder centerline remains consistent with the CFD results. Although the temporal resolution significantly affects the reconstruction accuracy of the modal coefficients, this influence is confined to the low-energy modes with indices greater than six. The minimal reduced-order model ensures an approximate reconstruction of the high-energy modal coefficients and, even at excessively coarse temporal resolutions, maintains a reasonable accuracy in the velocity field reconstruction.

**Figure 15:**
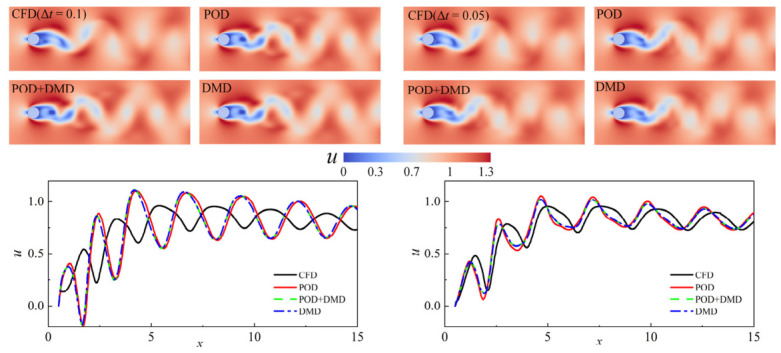
The effect of velocity field reconstruction of the ROM with six degrees of freedom at *t* = 198.

Figure 15 shows the velocity field reconstruction for a ROM consisting of modal coefficients (1, 2, 5, 6, 7, 8). When the time resolution is too large, the reconstructed velocity field looks very bad, and even the spatial distribution trend of the velocity is difficult to match. The main reason is that minor differences in manifold dynamics can seriously affect the accuracy of the reconstruction of the modal coefficients (4, 5, 6, 7), which in turn affects the accuracy of the reconstruction of the slaved modal coefficients. The minimal ROM avoids this risk, so the accuracy and stability of the ROM have absolute advantages in the case of insufficient time resolution.

When considering factors such as the accuracy of constructing manifold dynamics and the interpretability of ROM, POD + DMD modal coefficients are recommended, followed by DMD and POD modal coefficients. The dual effects of the energy and frequency of the POD + DMD mode are the keys to constructing a minimal and accurate ROM. The conclusions presented in this study are derived from the benchmark case of two-dimensional laminar flow past a circular cylinder in a microchannel at Re = 100. This flow demonstrates pronounced periodic behavior, characterized by a single vortex-shedding frequency and a low-dimensional attractor. Under these conditions, both DMD and POD + DMD effectively capture the dominant manifold dynamics. The primary advantages of POD + DMD are evident in its superior reconstruction accuracy for low-energy passive modes and its robustness to coarse temporal resolution. For more complex flows, such as the mixing of different concentrations in microchannels, the modal coefficients may not be reducible to single-frequency oscillations, and the spectral projection basis of POD + DMD may become invalid. In these scenarios, the conclusions of this study may not be applicable, and the method may require further modification or validation.

(3) Considerations regarding temporal resolution

In conventional flow analysis, adherence to the Nyquist–Shannon sampling theorem, which requires a sampling rate at least twice the highest frequency of interest, is typically sufficient for signal reconstruction. However, SINDy operates by reconstructing the underlying nonlinear governing equations rather than the signal itself. Even with adequate signal sampling, minor errors in numerical derivatives can be significantly amplified when incorporated into the high-order polynomial library (Figure 13 and Figure 15). As a result, the sparse regression algorithm may interpret numerical discretization noise as spurious nonlinear terms, potentially producing an incorrect (Equation (13)) or even divergent manifold dynamics equation. In this study, sampling rates of 600 times (Δ*t* = 0.01) or 120 times (Δ*t* = 0.05) the characteristic frequency are employed to reduce interference between temporal discretization errors of the dominant modes and the physical nonlinearities. This approach ensures that the identified equation is determined solely by the flow physics. This phenomenon is similar to that observed in macroscopic periodic chaotic systems, where SINDy continues to exhibit substantial generalization errors at low sampling rates [38], even when the Nyquist criterion is met.

While high temporal resolution enhances mathematical analysis, it introduces significant constraints in practical engineering and experimental contexts. For instance, in microchannel experiments (D = 100 μm), micro-particle image velocimetry necessitates a high-speed camera (frame rate exceeding 1 kHz) and a high-repetition-rate laser. Additionally, the response time of tracer particles and Brownian motion noise at the microscale restrict the effective sampling rate, and extended high-frequency acquisition can result in laser-induced thermal damage. Notably, high-energy dominant modes are relatively insensitive to temporal resolution, whereas passive modes (low-energy, high-order modes) require extremely high resolution for accurate full-field reconstruction. Therefore, if the engineering objective is limited to controlling the dominant vortex-shedding frequency, the sampling rate may be significantly reduced. Only when comprehensive and high-fidelity reconstruction of flow field details is necessary should the computational expense of a 120-times sampling rate be justified.

## 6. Conclusions

Based on the modal coefficients of POD, POD + DMD, and DMD, this study compares the differences between nonlinear correlation and the ROM constructed by the SINDy model. Without closure, the nonlinear dynamic system can be reduced to a ROM composed of two degrees of freedom by using the nonlinear correlation in the modal coefficients. The main conclusions are as follows:

(1) The ROM based on POD + DMD and DMD modal coefficients can accurately maintain the essential kinematic resolution and have a specific physical interpretation. Both of them get the same type of manifold dynamics and are expressed in the form of modal coefficients in pairs. The time derivatives of the real and imaginary parts of the modal coefficients are the imaginary and real parts of the corresponding modal coefficients. The absolute value of the coefficients is the fundamental frequency of vortex shedding. This characteristic is consistent with the sine and cosine functions in the trigonometric function, indicating that the modal coefficient’s phase difference between the real part and the imaginary part of the modal coefficient is π/2.
(2) The active modal coefficient is generally composed of the coefficient corresponding to the high-energy mode, and it is easy to find according to the distribution of RDC. Compared with STLSQ, SSR, and SR3 methods, the FROLS method is more suitable for constructing the nonlinear correlation of modal coefficients. It is easy to divide the nonlinear correlation into pure harmonics.
(3) The time resolution significantly impacts the construction of nonlinear correlation and manifold dynamics. For POD and DMD modal coefficients, slight differences in constructing manifold dynamics can lead to erroneous reconstructions of nonlinear dependencies. POD + DMD modal coefficients are highly recommended when considering factors such as the accuracy and interpretability of ROM.

## Figures and Tables

**Table 1:** Grid independence validation of the Strouhal number and the cumulative energy fractions of the first five POD modes.

Number of Cells	St Number	Cumulative POD Energy Fraction (%)
1.2 × 10^4^	0.179	89.9%
3.7 × 10^4^	0.176	87.5%
6.4 × 10^4^	0.178	85.4%
1 × 10^5^	0.171	84.8%
1.6 × 10^5^	0.172	85.1%

**Table 2:** Effect of temporal resolution on numerical convergence.

Δ*t*	St Number	Cumulative POD Energy Fraction (%)
0.1	0.171	84.1%
0.05	0.171	85.5%
0.01	0.172	85.1%

**Table 3:** Effects of different time resolutions on manifold dynamics.

Δ*t*	POD + DMD	DMD	Error
0.1	±1.061	±1.061	0.56%
0.05	±1.066	±1.066	0.09%
0.01	±1.067	±1.067	/

## Data Availability

The data that support the findings of this study are available from the corresponding author upon reasonable request.

## Abbreviations

ROM	Reduced-Order Model
POD	Proper Orthogonal Decomposition
DMD	Dynamic Mode Decomposition
ODE	Ordinary Differential Equations
EIM	Empirical Interpolation Method
DEIM	Discrete Empirical Interpolation Method
SINDy	Sparse Identification of Nonlinear Dynamics
CFD	Computational Fluid Dynamics
FVM	Finite Volume Method
RDC	Randomized Dependence Coefficient
PSD	Power Spectral Density
SSR	Stepwise Sparse Regressor
SR3	Sparse Relaxation Regularized Regression
FROLS	Forward Regression Orthogonal Least Squares
